# NMR Fragment-Based Screening against Tandem RNA Recognition Motifs of TDP-43

**DOI:** 10.3390/ijms20133230

**Published:** 2019-06-30

**Authors:** Gilbert Nshogoza, Yaqian Liu, Jia Gao, Mingqing Liu, Sayed Ala Moududee, Rongsheng Ma, Fudong Li, Jiahai Zhang, Jihui Wu, Yunyu Shi, Ke Ruan

**Affiliations:** 1 Hefei National Laboratory for Physical Sciences at the Microscale, School of Life Sciences, University of Science and Technology of China, Hefei 230027, China; 2 CAS, Center for Excellence in Biomacromolecules, Chinese Academy of Sciences, Beijing 100101, China

**Keywords:** epigenetics, protein-RNA interaction, RRM domain inhibitor, NMR fragment-based screening, TDP-43

## Abstract

The TDP-43 is originally a nuclear protein but translocates to the cytoplasm in the pathological condition. TDP-43, as an RNA-binding protein, consists of two RNA Recognition Motifs (RRM1 and RRM2). RRMs are known to involve both protein-nucleotide and protein-protein interactions and mediate the formation of stress granules. Thus, they assist the entire TDP-43 protein with participating in neurodegenerative and cancer diseases. Consequently, they are potential therapeutic targets. Protein-observed and ligand-observed nuclear magnetic resonance (NMR) spectroscopy were used to uncover the small molecule inhibitors against the tandem RRM of TDP-43. We identified three hits weakly binding the tandem RRMs using the ligand-observed NMR fragment-based screening. The binding topology of these hits is then depicted by chemical shift perturbations (CSP) of the ^15^N-labeled tandem RRM and RRM2, respectively, and modeled by the CSP-guided High Ambiguity Driven biomolecular DOCKing (HADDOCK). These hits mainly bind to the RRM2 domain, which suggests the druggability of the RRM2 domain of TDP-43. These hits also facilitate further studies regarding the hit-to-lead evolution against the TDP-43 RRM domain.

## 1. Introduction

RNA recognition motifs (RRMs) play diverse roles in post-transcriptional gene expression events such as RNA transport, localization, stability, and mRNA and rRNA processing. RRM is also known as the ribonucleoproteins (RNP) domain, as it contains the short and conserved elements RNP1 and RNP2, or RNA binding domain (RBD), that are abundantly distributed in higher vertebrates [1] and ubiquitously found in all kingdoms of life, including viruses and prokaryotes. In addition, they also participate in important functions such as microRNA biogenesis, apoptosis, and cell division [2,3]. RRMs are not only known to be involved in protein–nucleotide interactions, but also in protein–protein interactions [4].

The transactive response DNA-binding Protein 43kDa (TDP-43) is a RRM-containing protein, which plays important functions in mRNA metabolism regulation, including transcription repression, exon skipping, and RNA splicing [5,6]. TDP-43 is originally a nuclear protein, but translocates to the cytoplasm upon a pathological condition. It is a ubiquitously expressed, highly conserved, and multifunctional RNA and DNA-binding protein [7]. TDP-43 stabilizes the mRNA of human low-molecular-weight neurofilament (hNFL) [8]. Depletion of TDP-43 has important consequences in essential metabolic processes in human cells, like nuclear shape deformation, apoptosis, and misregulation of the cell cycle [9]. The disruption of TDP-43 auto-regulation impacts both localization of TDP-43 and its level, which results in TDP-43 accumulation in the cytoplasm. Based on its crucial roles in RNA processing, dysfunctional TDP-43 causes some abnormalities in alternative mRNA splicing, miRNA biogenesis, and RNA-rich granules formation [10].

The dysregulation of TDP-43 is hence associated with a variety of human diseases, especially neurodegenerative diseases, e.g., frontotemporal lobar degeneration (FTLD), amyotrophic lateral sclerosis (ALS), brain ischemia, aging, and Alzheimer’s disease [11,12,13]. For instance, in cases of FTLD and ALS, TDP-43 is the main constituent of their ubiquitin inclusions [14]. During the stress conditions, TDP-43 is localized in the cytoplasm, with mRNA binding to its RRM and glycine-rich domain, and thus forms the isolated liquid compartment enriching the mRNA and proteins. Such stress granules (SGs) in cells and in pathological brain tissue play crucial roles in FTLD/ALS pathology [15,16]. Aggregate-prone TDP-43 variants or exposure to oxidative stress generates distinct TDP-43 inclusions devoid of SGs [17]. The toxicity of the TDP-43 overexpression requires the presence of functional RNA Recognition motifs [18,19,20]. Recently, the proteinopathy of both important mutations (D169G and K263E located at RRM1 and RRM2, respectively) was computationally explored and the mutants are more prone to aggregation, causing neurological disorders [21].

Apart from the TDP-43 involvement in neurodegenerative diseases, an accumulating amount of evidence suggests that TDP-43 is a cancer responsive factor. TDP-43 positively contributes to the anticancer activity for curcumin in MCF-7 cells [22] and as a tumor suppressor by partnering with the TRIM16 in inhibiting the viability and proliferation of neuroblastoma and breast cancer cells [23]. In addition, normal levels of TDP-43 might be a crucial protective factor for cells under apoptotic insult [24]. On the contrary, the TDP-43 inhibition suppressed cervical cancer cell growth and induced cell cycle arrest while its overexpression promoted cancer cell progression and drove the cell cycle [25]. TDP-43 may regulate melanoma cell proliferation and metastasis by modulating glucose metabolism [26]. TDP-43 also plays an oncogenic role in malignant glioma cell progression by stabilizing small nucleolar RNA host gene 12 (SNHG12) [27]. The findings demonstrated that TDP-43 regulates the MALAT1, a non-coding RNA overexpressed in non-small cell lung cancer (NSCLC), through direct binding to MALAT1 RNA at the 3′ region by RRM, whose participation is compulsory. This controls the growth, invasion, and migration of NSCLC cells [28]. Reduced tumor progression, including proliferation and metastasis, was observed upon the knockdown of TDP-43 in triple-negative breast cancer (TNBC) and RRM involvement is assured [29]. These studies suggest that targeting the TDP-43 RRM domains may, therefore, be an effective therapeutic approach for neurodegenerative diseases and cancers.

Although more is known about the TDP-43 biology and its association with neurodegenerative and cancer diseases, the development of treatments toward TDP-43 is mostly lagging behind those targeting other proteins involved in such diseases [30]. RRM and RNA complexes have long been attractive targets for small molecule inhibition targeting the RNA, not the protein [31,32]. Firstly, the aminoacridine derivative was discovered to interrupt the formation of RNA and U1A RRM1 complex [33]. Additionally, a high-throughput screening assay, based on AlphaScreen^®^, technology was used to characterize DNA and RNA oligonucleotides (bt-TAR-32 and bt-TG6, respectively) binding to TDP-43 and their interaction inhibition was assessed [34]. Later, that series of 4-aminoquinoline derivatives were characterized for their capacity to modulate TDP-43 metabolism and function, whereby they bind to TDP-43, reduce its interaction with the oligonucleotide, and stimulate caspase-mediated cleavage of TDP-43 [35], but information is still lacking on the binding topology. Furthermore, some medicinal treatment reduces the TDP-43 inclusions through the autophagy pathway were discussed [36]. However, no compounds directly targeting RRM domains of TDP-43 have been uncovered to our best knowledge.

NMR spectroscopy is a powerful approach which has been extensively used by the pioneers in fragment-based drug discovery for detecting molecular interactions between the target and the fragment libraries [37,38,39] and to facilitate structure-based drug design [40]. Consistently, the fragment-based screening approach has been fruitful for identifying hits for the challenging protein-protein interaction “hot-spots” [41,42,43,44,45]. We expect it shall be effective in the case of the shallow RNA binding pocket of TDP-43 tandem RRMs.

Here, we carried out automated NMR fragment-based screening [46] to identify three hits of the tandem RRMs of TDP-43. Chemical shift perturbations of the ^15^N labeled TDP-43 tandem RRMs demonstrate that these hits bind to the same site, mainly on the RRM2 domain. It has also been validated by the chemical shift perturbation experiments for TDP-43 RRM2 alone. The CSP-driven HADDOCK was used to generate the protein-hits binding mode. Collectively, our work provides a class of compounds for further hit-to-lead evolution of the TDP-43 RRM domain and paves the path for targeting protein-RNA interactions using the fragment-based approach.

## 2. Results

Structurally, TDP-43 tandem RRMs are approximately 160 amino acids long and display a β1α1β2β3α2β4 arrangement of secondary structure, with an additional β-hairpin named β3’β3’’ [47] or β5 [48,49] which is located between α2β4, and extends the β-sheet surface to be accessible to binding by multiple RNA nucleotides. This leads to a rare RRMs orientation type (β2β4) and the 14-aa linker needs to connect four β-strands instead of two [2,47]. Diverse studies revealed that TDP-43 tandem RRMs can interact with both short and long single-stranded nucleic acids rich in UG/TG, either separately or collectively, to achieve high affinity and specificity [47,48,49]. Given the RNA recognition mode by tandem RRMs, TDP-43 RRMs are independent of each other in unbound form but they establish a rigid structure upon RNA binding on the flat surface β-sheet [47]. In general, this RNA-recognition pocket is much shallower than the ATP-binding sites of kinases. Hence, it poses a grand challenge for conventional high throughput screening aimed at discovering strong binders. Conversely, the fragment-based approach has proven fruitful for uncovering the initial hits, albeit at weak affinities.

**Figure 1:**
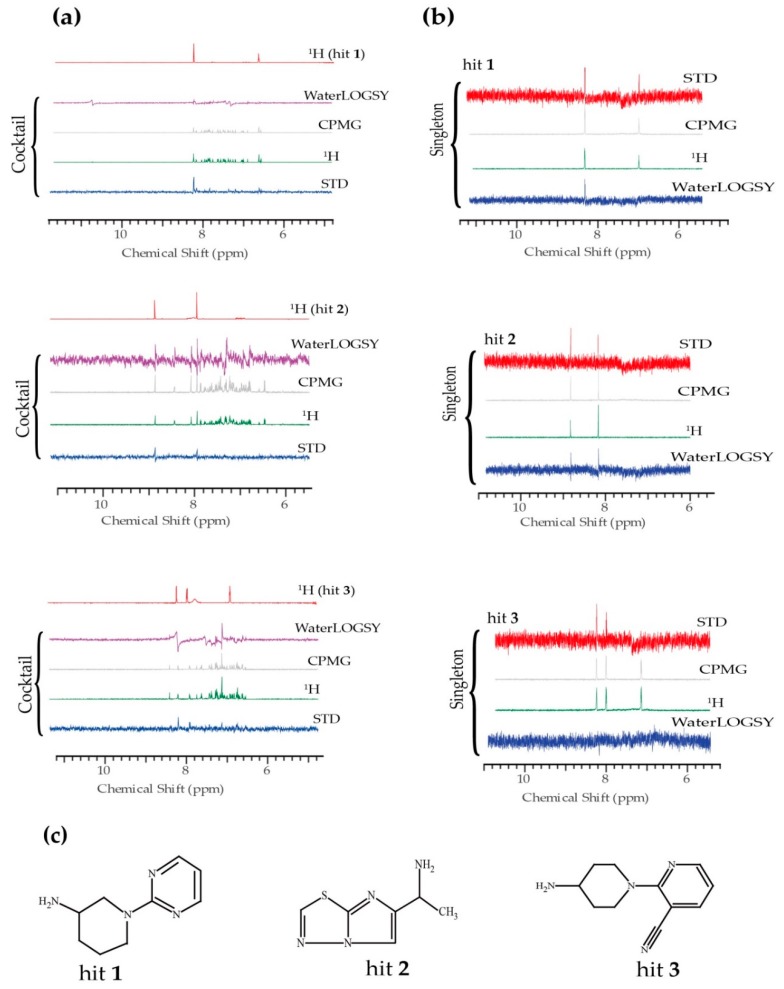
NMR fragment-based screening against the tandem RRM domain of TDP-43. (**a**) The primary screening WaterLOGSY, CPMG, ^1^H and STD spectra for three representative cocktails. The ^1^H reference spectrum of the respective hit is shown for comparison. (**b**) The secondary screening spectra for individual hit 1, 2, and 3, respectively. (**c**) The chemical structures of hits 1, 2, and 3.

NMR ligand-observed methods detect the weak protein-ligand binding by detecting changes in the characteristics of the ligand spectrum that occur upon binding to the protein. Using the ligand-based experiments, i.e., saturation transfer difference (STD) [50], water ligand observed via gradient spectroscopy (WaterLOGSY) [51], Carr–Purcell–Meiboom–Gill (CPMG) [52], and ligand-based 1D proton, we found 17 hits from the primary screening of 89 cocktails containing 10 compounds each (Figure 1a). The binders present signals while the non-binders present no signals in the STD spectra. Accordingly, the binders show inverted or a fast decay of signals in the WaterLOGSY and CPMG experiments, respectively. The combined output of these spectra enabled the identification of primary screening hits from cocktails. It is worth noting that the reference 1D proton spectra of each individual compound might be slightly different from the screening spectra as a different buffer was used to be better compatible with TDP-43 tandem RRMs. The primary screening hits were further validated by the secondary screening for individual hits using the same set of NMR experiments (Figure 1b and Figure S1). The aromatic peaks of the hit are depicted as they suffer less from the interference of buffer signals. The secondary screening eliminated 13 primary hits, probably due to sample aggregation in cocktails, ambiguous selection of hits with degenerated chemical shifts, and/or spectrometer instability. Among the remaining 4 hits, hit 2 demonstrated a distinct topology relative to hits 1 and 3 (Figure 1c).

**Figure 2:**
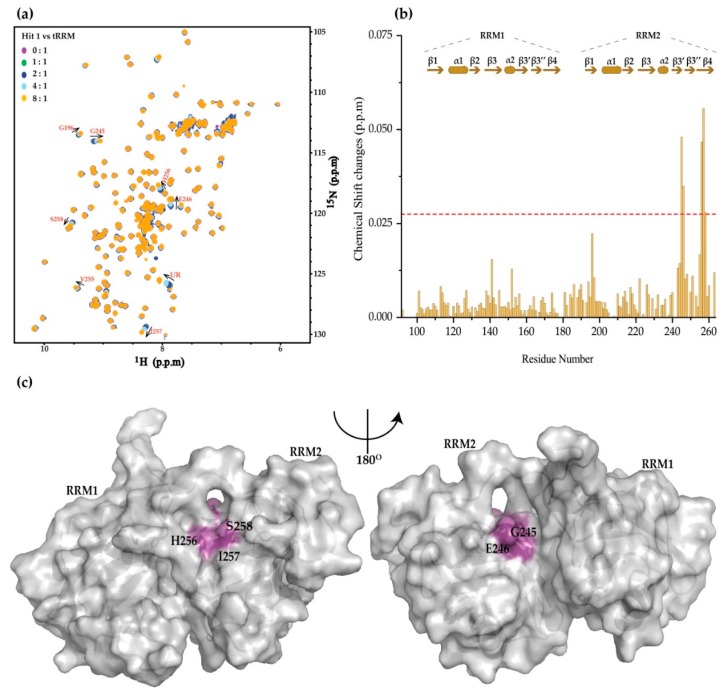
The binding topology of hit 1 on the tandem RRMs of TDP-43 using NMR chemical shift perturbations. (**a**) The chemical shift perturbations of ^15^N-labeled tandem RRM domain of TDP-43 upon titration of hit 1. The ligand/protein molar ratios are annotated. The perturbed residues are labeled and the arrows indicate the direction of chemical shift changes. UR stands for unassigned residue. (**b**) Chemical shift changes of the TDP-43-tandem RRM domain are at the ligand protein molar ratio of 8:1. The red horizontal dashed line represents two standard deviations above the averaged chemical shift changes of residues. (**c**) Surface representation of TDP-43 tandem RRM domain (PDB code: 4BS2) showing the purple-colored residues with significant chemical shift changes.

**Figure 3:**
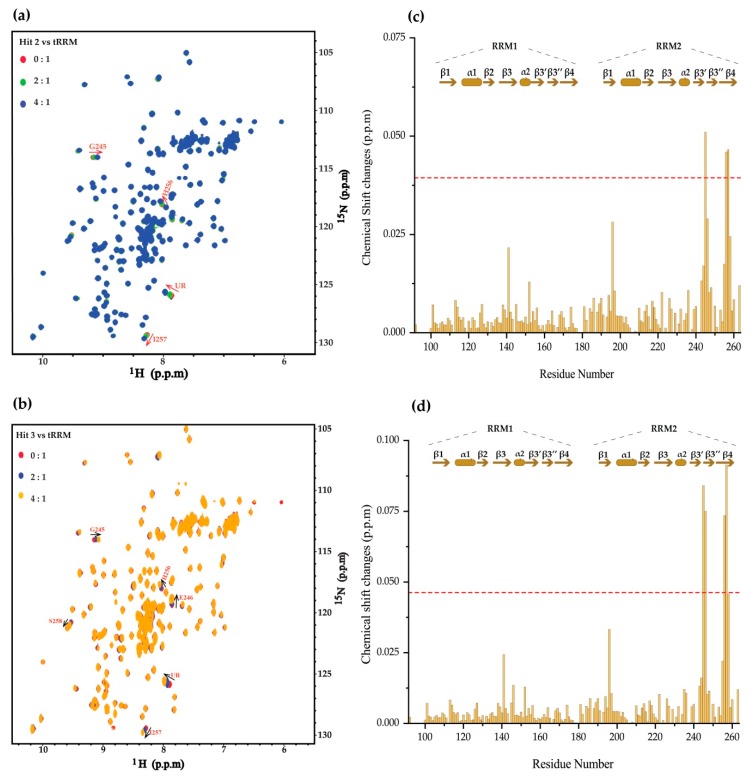
Chemical shift perturbations of tandem RRM upon binding of hit 2 and 3. (**a**,**b**) The chemical shift perturbations of TDP-43 tandem RRM domain induced by titration of hit 1 and 2, respectively. Annotated are the hits: Protein molar ratios. UR stands for unassigned residue. (**c**,**d**) Residue-by-residue chemical shift changes of tandem RRM at the hit/protein molar ratio of 8:1 for compound 2 and 3, respectively. The red dashed lines represent two standard deviations above the averaged chemical shift changes of residues.

The 4 secondary screening hits were then cross-validated using the chemical shift perturbations (CSPs) of the ^15^N-labeled tandem RRMs of TDP-43 and 3 of them induced significant chemical shift changes of the tandem RRM (Figure 2 and Figure 3). This approach has been extensively applied in the interrogation of protein-ligand interactions in an affinity ranging from nM to mM. As CSP is a sensitive indicator of chemical environment changes induced by ligand titration, it is particularly powerful in the detection of weak bindings. The linewidths of the amide signals of TDP-43 tandem RRM show almost no changes upon titration of hit 1 (Table S1), which suggests that hit 1 induces no protein aggregation. This is a useful approach to remove false positives, which are commonly found in drug screening because of protein aggregation [53]. Titration of hit 1 induces dose-dependent CSPs of residues G245, E246, H256, I257, S258 (Figure 2b and Figure S2). However, the curve does not reach the saturation point, as it is limited by the weak binding affinity and the low aqueous solubility of the hit. Hence, the binding affinity of those weak binders cannot be robustly estimated from CSPs. The disturbed residues were then mapped on the surface representation of the solution structure of TDP-43 tandem RRMs (PDB code: 4BS2) [47]. Residues H256, I257, S258 locate on the β4 strand, while residues G245 and E246 bridge the α2 and β3 (Figure 2c).

Consistently, hits 2 and 3 titrations also point to the same binding topology in the tandem RRM of TDP-43 (Figure 3). For example, hit 2 perturbed residues G245, H256, and I257 (Figure 3a,c), while hit 3 induced significant CSPs for residues G245, E246, H256, and I257 (Figure 3b,d). The similarity of the binding pattern of the three hits suggests that weak but specific binders were successfully identified using the NMR fragment-based screening.

**Figure 4:**
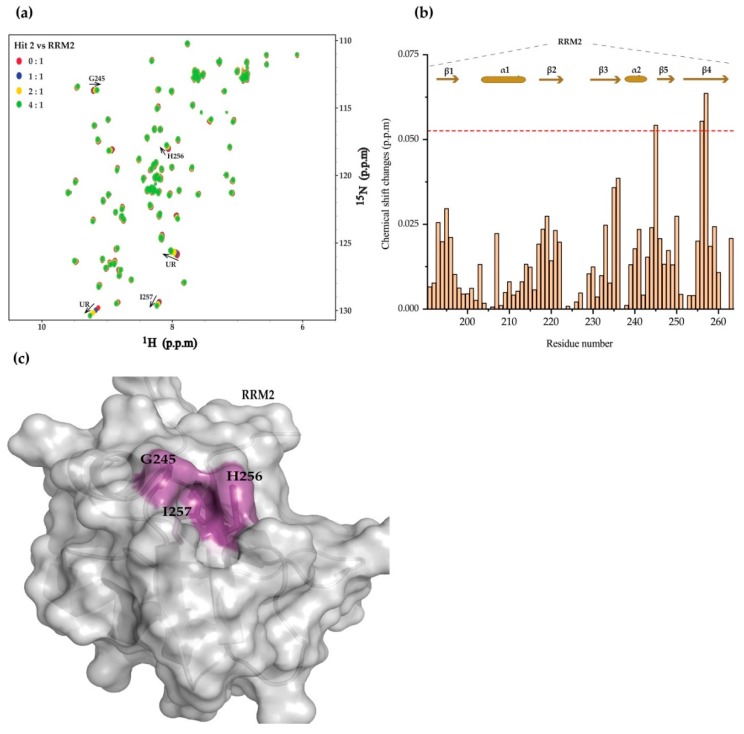
Chemical shift perturbations of the TDP-43-RRM2 domain upon hit 2 titration. (**a**) The chemical shift perturbations of the RRM2 domain of TDP-43 by hit 2 titration. (**b**) Chemical shift changes of the TDP-43 RRM2 domain residues at a hit 2; protein molar ratio of 4:1. The red dashed line represents two standard deviations above the averaged chemical shift changes of residues. (**c**) Residues (colored in purple) undergo significant chemical shift changes and are mapped on the surface representation of TDP-43-RRM2 domain (PDB code: 1WF0).

Having confirmed that 3 different hits bind on the same site of the TDP-43 RRM2 domain, we further investigated whether RRM2 alone is sufficient for ligand binding. Hit 2 was thus titrated to the ^15^N-labeled RRM2 domain of TDP-43 (Figure 4a). Consequently, the residues G245, on loop bridging the α2 and β3′, H256, and I257, located on β4-strand, were perturbed (Figure 4b). Those residues were mapped on the surface representation of the TDP-43 RRM2 [49] domain in complex with a single-stranded DNA (Figure 4c). The hit binds to the same sites of either TDP-43 tandem RRM or RRM2 alone. That is to say, TDP-43 RRM2 is the main contributor for ligand binding and should be considered as the target for follow-up hit-to-lead evolutions.

**Figure 5:**
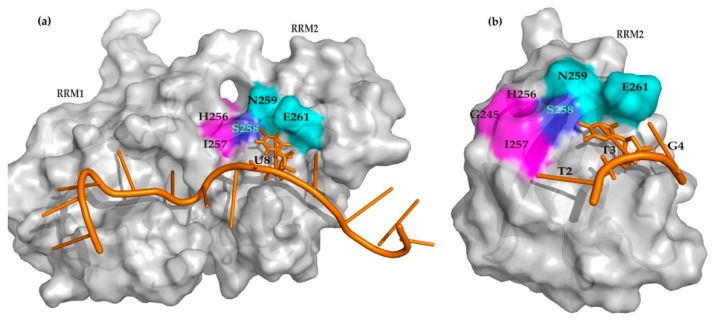
Comparison of binding sites of nucleic acids and hits on TDP-43. (**a**) Surface representation of TDP-43 tandem RRMs in complex with AUG12 RNA (orange cartoon), where residues interact with the U_8_ nucleotide (stick) and hits are highlighted in cyan and magenta, respectively. Residue S258 (blue) interacts with both U_8_ and hit 1. (**b**) Surface representation of TDP-43 RRM2 in complex with ssDNA (PDB code: 3D2W) using the same coloring scheme.

We further compared the small molecule binding topology with the nucleic acid recognition sites of the TDP-43 RRM domain. In TDP-43 tandem RRMs, 10 out of 12 nucleotides of the AUG12 RNA (GUGUGAAUGAAU) interact with RRM1 and RRM2 (PDB code: 4BS2) [47]. Among them, the first five (G_1_U_2_G_3_U_4_G_5_) nucleotides are accommodated on the RRM1 β-sheet and the following two nucleotides (A_6_A_7_) act as a connector between two RRMs, while the next three nucleotides (U_8_G_9_A_10_) lie on the RRM2. The U_8_ nucleotide of RNA is recognized on S258 (β4) through hydrogen bonds, on the backbone carbonyl oxygen of N259 (β4), and the backbone amide of E261 from the C-terminus [47]. Comparatively, all three hits have perturbed some residues located on the β4-strand, hits 1 and 3 specifically disturbed S258 (β4). This also interacts with the U_8_ nucleotide in tandem RRM (Figure 5a). Furthermore, the RRM2 residues D247 (loop α2-β3′) and I249 (β3′) are involved in inter-RRM interactions upon RNA binding on the tandem RRM of TDP-43. This study revealed that their nearby residues, G245 and E246 (loop α2-β3′), display higher chemical shift perturbations induced by the hits binding (Figure 2b and Figure 3c,d).

Accordingly, the crystal structure of TDP-43 RRM2 in complex with ss-DNA 5′-GTTGAGCGTT-3′ (PDB entry: 3D2W) reveals that only three 5′ end nucleotides (T2, T3, G4) make extensive contacts with β-sheet residues of RRM2, whereby T3 particularly contacts with S258, Asn259, and Glu261 through hydrogen bonds [49], while in our study the residues H256 and I257, nearby the S258 (β4), have been perturbed upon hit binding on the single RRM2 (Figure 5b). This suggests that the fragment screening hits bind to a proximal site for RNA/DNA recognition, thus new hits can be designed using a fragment grow strategy to block the DNA/RNA recognition capability of TDP-43 RRM2.

**Figure 6:**
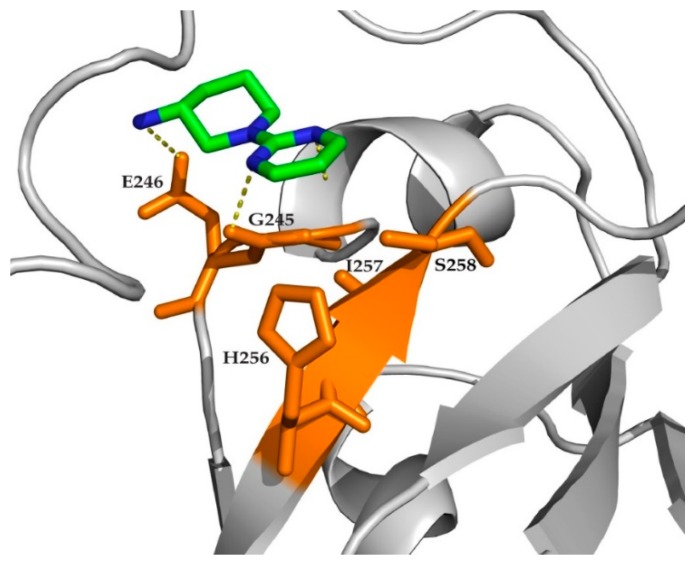
The representative docking model of hit 1 in consistency with experimental CSP and STD restraints. Hit 1 (green color) in the binding site of tandem RRM (PDB: 4bs2) where the carbonyl hydrogen is oriented toward G245, while the side chain hydrogen interacts with E246 residue of tandem RRM. Other active residues (orange sticks), H256, I257, and S258 are located in proximal of the hit 1.

To further characterize the binding mode, a data-driven approach, HADDOCK [54], was used to model the tandem RRM-hit 1 complex structure. Residues G245, E246, H256, I257, and S258 were defined as active ones in the binding site. Among the docking poses generated by HADDOCK, the best-fit ones were filtered out based on CSP and STD restraints [41,55,56]. One representative docking pose (Figure 6) indicates that hit 1 forms a hydrogen bond with the side chain of S258 and the aromatic ring of hit 1 is proximal to residues G245, E246, H256, and I257. These docking poses pave the path for following structure-guided hit-to-lead evolution.

## 3. Discussion

Proteins containing RRM domains function in important aspects of the posttranscriptional regulation of gene expression, mRNA maturation, and other RNA processing machinery. These proteins perform their diverse roles depending on the dual ability to recognize RNA and to interact with other proteins by using their RRM domain [31]. As TDP-43 is closely correlated with neurodegenerative and cancerous diseases [29,57], the RRM domain of TDP-43 becomes an attractive therapeutic target. However, there is no direct inhibitor targeting the RRM discovered to date.

We uncovered three small molecules binding to the tandem RRM domain of TDP-43 by using NMR fragment-based screening techniques. The NMR spectroscopy, one of a plethora of biophysical methods, is particularly powerful to detect even ultra-weak protein-ligand interactions. Accordingly, chemical shift perturbations observed in the heteronuclear single-quantum coherence (HSQC) spectra or the linewidth analysis of the small molecules allow the determination of binding affinity [58,59]. This is sometimes recalcitrant, as the titration to saturation point may be infeasible in case of weak binding affinities and low aqueous solubility of compounds.

NMR is extensively applied in fragment-based lead discovery [60]. The central idea is to screen a small library (500–2000 molecules) of low-molecular-weight compounds (110–250 Da), as their low complexity enhances the probability of matched interactions between the target and these fragment compounds. The reasonable hit rate indicates the druggability of the TDP-43 tandem RRM domain.

Although the 4-aminoquinolines molecules have been discovered through high throughput screening against the full-length TDP-43 [34], the enlightenment on binding site is still lacking. TDP-43 contains two RNA-binding RRM domains and the C-terminal low complexity domain, which may form liquid–liquid phase separation as a reservoir of mRNAs. Here, it is essential to determine the small molecule binding topology on TDP-43. The tandem RRM of TDP-43 is composed of a canonical RRM arrangement (β1α1β2β3α2β4), with an additional β-hairpin (β3’β3’’ or β5) found between α2 and β4 which extends the β-sheet surface for RNA recognition [2,47,49]. The binding topology of our fragment screening hits and CSP-guided HADDOCK modeling reveal a ligand-binding “hot spot” of TDP-43 RRM2, proximal to H256, I257, and S258. Interestingly, these residues are also close to the RRM1 and RRM2 interface. The previous study proposed that both RRM domains are indispensable for achieving the greater binding affinity between the TDP-43 and nucleic acids [49]. Since this “hot spot” is partially overlapped with the RNA/DNA recognition site, it directs the following structure-guided hit-to-lead evolution against TDP-43 tandem RRM domains.

## 4. Materials and Methods

### 4.1. Cloning, Expression, and Protein Purification

The tandem RRM domain of TDP-43 (residues 101–269) was synthesized by GENEWIZ (Suzhou, China) and sub-cloned into the pET22b vector (GE Healthcare, Shanghai, China) with the His_6_ tag. The RRM2 domain was amplified from the tandem RRM construct and then sub-cloned into the pET22b vector (GE Healthcare, Shanghai, China) with the His_6_ tag. The constructs were transformed into *Escherichia coli* BL21 and cultivated in 1 L LB media, incubated at 37 °C. The proteins were expressed at 16 °C after induction by 0.5 mM isopropyl β-D-thiogalactosidase (IPTG) for 20 h. The bacteria were harvested by centrifugation (5000 rpm, 10 min), resuspended in lysis buffer (25 mM Tris, 500 mM NaCl at pH 7.5), and then lysed by sonication. The cell lysates were centrifuged (13,000 rpm, 30 min). The collected supernatant was purified on a column filled with Nickel-chelated resin (QIAGEN, Shanghai, China). The impurities were washed out using a buffer (25 mM Tris, 1 M NaCl at pH 7.5) containing a linear gradient of 20–40 mM imidazole, then the same buffer containing 500 mM imidazole was used to elute out the target proteins. All proteins were further purified by size exclusion chromatography using a HiLoad 16/600 Superdex 75 column (GE Healthcare, Shanghai, China). The target proteins were confirmed by SDS-PAGE.

For ^15^N-labeled proteins, the cells were first cultured in 1 L LB media, harvested when A_600_ reached 1.0 and then transferred to 1 L M9 media containing ^15^NH_4_Cl. The cells were induced by 0.4 mM IPTG to express the proteins (tandem RRMs and RRM2 domains). The purified proteins were concentrated in PBS buffer plus the 5 mM DTT at pH 7.5.

### 4.2. NMR Fragment-Based Screening

All NMR fragment screening experiments were carried out at 25 °C using an Agilent 700 MHZ spectrometer equipped with a 96 well auto-sampler and a 5 mm cryoprobe. During the primary screening, the ligand-based NMR spectra (STD, WaterLOGSY, CPMG, and 1D ^1^H) were acquired against the 890 fragments library (ChemBridge, San Diego, CA, USA) as described previously in detail [46,61]. Those fragments were distributed in 89 cocktails, composed of 10 compounds each, at a final concentration of 0.4 mM. These cocktails were incubated with protein (10 µM) in sodium phosphate (50 mM, pH 7.5), NaCl (200 mM), dithiothreitol (5 mM), and D_2_O (50%). To further confirm the identified primary hits, secondary screening was individually carried out for single hits using the same buffer and NMR experimental settings. We then automatically processed and visualized the primary and secondary data with our ACD/Labs scripts, as previously described [46].

### 4.3. NMR Chemical Shift Perturbation

NMR HSQC spectra were acquired at 25 °C on either an Agilent 700MHZ spectrometer equipped with a cryoprobe or an Agilent 500MHz spectrometer equipped with a room temperature probe. The ^15^N-labeled proteins (0.1 mM or 0.2 mM), in PBS buffer (50 mM, pH 7.5), containing NaCl (200 mM), dithiothreitol (5 mM), and D_2_O (10%) were titrated by small molecules stocked in DMSO at a concentration of 200 mM, using a series of hit/protein molar ratios of 0.0, 0.5, 1.0, 2.0, 4.0, and 8.0 for TDP-43 tandem RRMs and 0.0, 0.5, 1.0, 2.0, and 4.0 for RRM2, respectively. Spectra were processed in NMRpipe and analyzed with Sparky. The chemical shift changes (∆δ) relative to the free form of protein were defined as follows:

(1) $$\Delta \delta =\sqrt{{\left(\delta_{1_{H}}\right)}^{2}+{\left(0.2\delta_{15_{N}}\right)}^{2}},$$

where *δ*^1^_H_ and *δ*^15^_N_ are the chemical shift differences of the ^1^H and ^15^N dimensions, respectively. We referred to the following chemical shift assignments previously deposited in the Biological Magnetic Resonance Data Bank: RRM1 (BMRB Entry 18765), RRM2 (BMRB Entry 19922), and tandem RRM (BMRB Entry 19290). All structures figures were prepared by Pymol (DeLano Scientific, LLC, Palo Alto, CA, USA).

### 4.4. Molecular Docking

HADDOCK is an information-driven docking technique used for modeling biomolecule structures by using experimental or predictive restraints [54,62]. The CSPs, obtained from the NMR HSQC titration data, were used both as HADDOCK restraints and for defining the protein active residues. The tandem RRM structure (PDB: 4bs2) served as the starting structure, while the hit **1** PDB file was generated by the PRODRG [63]. The docking calculations were done by the HADDOCK web server and clustered 186 structures in 16 clusters according to the RMSD threshold of 2 Å.

### 4.5. Linewidth Analysis

The NMR HSQC spectra at molar ratios of 0:1 and 8:1 (hit/protein) were processed using the same NMRpipe script, e.g., 2-fold zero-filling, Fourier transformation, and phase corrections. The spectra were then analyzed, with randomly selected peaks, using Sparky. After peak integration, the linewidth, i.e., the full width at half the peak height, was automatically estimated by Sparky.

## Supplementary Materials

Supplementary materials can be found at https://www.mdpi.com/1422-0067/20/13/3230/s1.

## Abbreviations

ALS	Amyotrophic lateral sclerosis
ATG7	Autophagy-related protein 7
BMRB	Biological Magnetic Resonance Data Bank
CPMG	Carr–Purcell–Meiboom–Gill
CSPs	Chemical shift perturbations
FBS	Fragment-based screening
FTLD	Frontotemporal lobar degeneration
HADDOCK	High Ambiguity Driven biomolecular DOCKing
HDAC6	Histone deacetylase 6
hNFL	human low molecular weight neurofilament
HSQC	Heteronuclear single-quantum coherence
ITC	Isothermal titration calorimetry
NMR	Nuclear Magnetic Resonance
RNP	Ribonucleoproteins
RRM	RNA Recognition Motifs
SGs	Stress granules
SNHG12	Small nucleolar RNA host gene 12
SPR	Surface Plasmon Resonance
STD	Saturation Transfer difference
TDP-43	Transactive response DNA-binding Protein 43
TNBC	Triple-negative breast cancer
TRIM16	Tripartite motif-containing protein 16
WaterLOGSY	Water ligand observed via gradient spectroscopy
