# Supplementary material for: NMR Fragment-Based Screening against Tandem RNA Recognition Motifs of TDP-43
Source: Int J Mol Sci. 2019 Jun 30;20(13):3230. doi: 10.3390/ijms20133230 (PMC6651732; doi:10.3390/ijms20133230)
Supplement: Supplementary file 1 [file ijms-20-03230-s001.pdf]

Supplementary materials

Table S1. NMR linewidth (lw) analysis of ^{15}N labeled TDP-43 tandem RRM upon addition of hit **1**.

Residue	Free		+ hit **1***	
	lw^{15}N (hz)	lw^{1}H (hz)	lw^{15}N (hz)	lw^{1}H (hz)
**D236**	12.9	29.0	12.6	29.9
**F124**	11.4	27.4	11.2	27.5
**E156**	12.1	24.5	12.5	23.8
**F211**	13.0	28.1	12.9	28.1
**G245**	12.3	30.0	12.0	30.3
**G196**	11.5	29.9	11.4	30.8

* At the hit/protein molar ratio of 8:1.

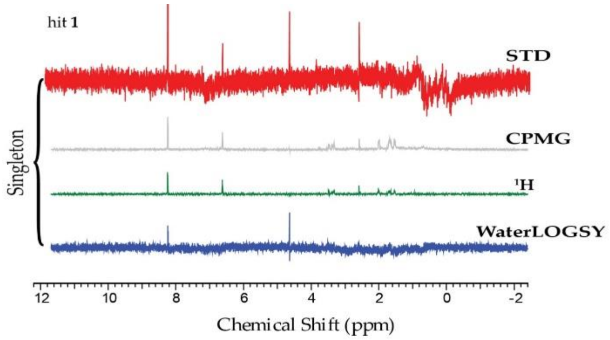

Figure S1. The whole NMR spectra of secondary screening for hit **1**.

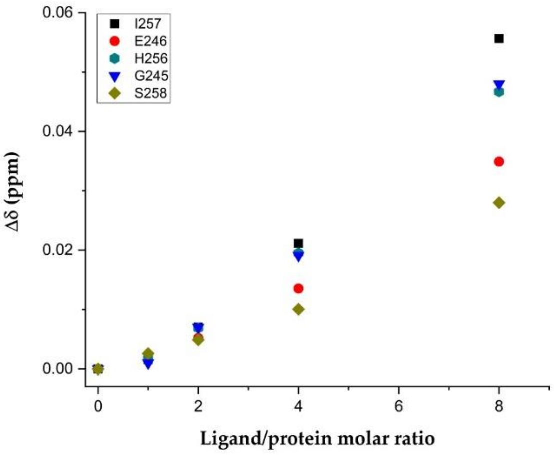

Figure S2: The dose-dependent titration curve for the tandem RRM disturbed residues upon hit **1** titration at different ligand/protein molar ratios: 0:1, 1:1, 2:1, 4:1, 8:1. Annoted are the disturbed residues. The curve does not reach the saturation point, probably due to the weak binding affinity of the hit **1**. Further titration of the small molecule is not feasible because of the low aqueous solubility of the compound. Therefore, the binding affinity of those weak binders cannot be robustly estimated from CSPs.
